# Association between Dry Eye Disease and Psychological Stress among Paramedical Workers in Korea

**DOI:** 10.1038/s41598-019-40539-0

**Published:** 2019-03-07

**Authors:** Joon Young Hyon, Hee Kyung Yang, Sang Beom Han

**Affiliations:** 1 Department of Ophthalmology, Seoul National University Bundang Hospital, Seoul National University College of Medicine, Seongnam, Korea; 2 Department of Ophthalmology, Kangwon National University Hospital, Kangwon National University Graduate School of Medicine, Chuncheon, Korea

## Abstract

This study was performed to evaluate the prevalence and risk factors of dry eye disease (DED) among paramedical workers at a university hospital in Korea. This cross-sectional study included 566 paramedical workers at a university hospital in Korea. Dry eye symptoms were assessed using a 9-item questionnaire, and DED was defined as having 1 or more dry eye symptoms often or all the time. A survey including demographic data and potential risk factors of DED was also performed. Psychological stress was measured using stress VAS and perceived stress scale 4 (PSS-4) questionnaires. Of the 566 paramedical workers, 232 (35 male and 197 female) completed the survey. Prevalence of DED was 42.7% (99/232). Univariate analysis revealed that female sex (*P* < 0.001), prolonged computer use (*P* = 0.003) and higher stress VAS (*P* < 0.001) and PSS-4 (*P* = 0.009) scores had significant association with DED. In multivariate analysis, DED had significant association with female sex (*P* = 0.003) and stress VAS (*P* = 0.013) after adjustment for sex, duration of computer use and stress VAS, and had significant association with female sex (*P* = 0.003) and durations of computer use (*P* = 0.029) after adjustment for sex, duration of computer use and PSS-4 score. In conclusion, DED was prevalent among paramedical workers in Korea. Its risk increased among females and workers with increased psychological stress. Prolonged use of computer was possibly associated with DED.

## Introduction

Dry eye disease (DED) is one of the most common diseases with a prevalence of 10–30% worldwide^1,2^. It has emerged as a major public health problem because the condition poses considerable amount of economic burden both to affected individual and society^3,4^. The symptoms of DED, such as, ocular discomfort, pain, grittiness, redness, dryness, foreign body sensation and visual disturbance can interfere with daily activities including reading, driving, using computer and watching TV^5,6^; thus, the disease can result in serious impairment of quality of life^4–6^.

DED is a multifactorial disease that is characterized by discrepancy between dry eye symptoms and ocular surface signs, which renders the diagnosis and management of the disease difficult^2,7–9^. Previous studies suggested that subjective dry eye symptoms can be influenced by individual pain perception or psychosomatic conditions including depression, anxiety and stress^10,11^. Increased use of video terminal display (VDT) is also shown be associated with the risk of DED^6,12^.

A large number of studies have been conducted to evaluate the nature of DED. However, there are only few data about the characteristics of DED in healthcare personnel^13,14^. Hospital workers are exposed to work requiring great concentration and large amount of VDT work in relatively less humidified environment in ward or operation room, which may increase the risk of developing the dry eye symptoms. However, to the best of our knowledge, there has been no report on nature of DED in hospital workers in Korea.

Therefore, in the present study, we evaluated the prevalence and risk factors of DED among paramedical workers at a university hospital in Korea.

## Patients and Methods

### Study Design and Population

This is a cross-sectional study that included 566 paramedical workers including nurse and medical technicians at Kangwon National University Hospital in Korea.

Only workers who fully understood the nature of the survey and agreed to participate were included. Exclusion criteria included active ocular surface inflammation, such as, infectious keratitis, infectious or allergic conjunctivitis, glaucoma, connective tissue disorders, history of ocular trauma, history of ocular surgery other than refractive surgery, systemic vasculitis.

Every participant was contacted by trained interviewers and requested to fill out a survey that comprises three categories of questions, as follows: (1) questions regarding dry eye symptoms, (2) questions about demographic data and potential risk factors of DED and (3) questions regarding psychological stress.

First, a Dry Eye Questionnaire (DEQ) was administered. The DEQ, which consisted of 9 questions pertaining to dry eye symptoms, was generated by modifying dry eye symptom questions suggested in the literature (Table 1)^8–10^. Each participant was asked to indicate whether each of the 9 symptoms related to DED was experienced rarely, sometimes, often, or all the time. The diagnosis of DED was made when a participant reported to have 1 or more symptoms often or all the time during the past 2 weeks.

**Table 1:** Questionnaire for dry eye symptoms in the present study.

1. Do your eyes feel dry?
2. Do you feel gritty or sandy sensation in your eyes?
3. Do your eyes ever have a burning sensation?
4. Do your eyes ever feel sticky?
5. Do your eyes ever feel watery or tearing?
6. Are your eyes ever red?
7. Do you notice much crusting or discharge on your lashes?
8. Do your eyes get stuck shut in the morning?
9. Have you experienced transient blurry vision?

Allowable responses to the questions were ‘none’, ‘rarely’, ‘sometimes’, and ‘often or all the time’. Dry eye disease was defined as having one or more symptoms often or all the time.

The dry eye visual analog scale (VAS) and ocular surface disease index (OSDI) questionnaires were also administered for the quantification of the dry eye symptoms, according to the literature^13,15–17^. The VAS questionnaire comprised three questions, each of which had an answer scale from 0 (no symptom) to 10 (the worst symptom imaginable), for pain, dryness and foreign-body sensation, respectively; thus, the total VAS score ranged from 0 to 30^16,18^. The OSDI questionnaire consisted of 12 questions related to dry eye symptoms that each subject experienced during one week prior to the interview^13,15–17^. The OSDI was expressed as a sum score of 0–100, according to the literature^13,15–17^.

Second, the participants also filled in the questionnaire regarding demographic data, such as, age by decades and sex, potential risk factors of DED including contact lens (CL) use, history of refractive surgery, hours of computer use, hours of paper work, sleep duration and personal habits including alcohol consumption. Age of each participant was collected by decade as per the recommendation from the IRB to prevent disclosure of identification of the participants.

Third, psychological stress was evaluated using the Perceived Stress Scale 4 (PSS-4) questionnaire and stress VAS, according to the literature^19–22^. PSS-4 questionnaire consisted of 4 questions pertaining to perceived psychological stress (Table 2), and used to quantify the perceived stress between the score of 0 to 16, with higher scores reflected an increased perceived psychological stress^22^. Stress VAS score was also used for the measurement of psychological stress, with an answer scale from 0 (no stress) to 10 (extreme stress that can be imagined)^19–21^.

**Table 2:** Perceived Stress Scale 4 (PSS-4).

1. In the last month, how often have you felt that you were unable to control the important things in your life?
2. In the last month, how often have you felt confident about your ability to handle your personal problems?
3. In the last month, how often have you felt that things were going your way?
4. In the last month, how often have you felt difficulties were piling up so high that you could not overcome them?

Allowable responses to the questions were ‘never’, ‘almost never’, ‘sometimes’, ‘fairly often’ and ‘very often’.

Scoring for the PSS-4 is as follows:

Questions 1 and 4, 0 = never; 1 = almost never; 2 = sometimes; 3 = fairly often; 4 = very often.

Questions 2 and 3, 4 = never; 3 = almost never; 2 = sometimes; 1 = fairly often; 0 = very often.

### Statistical analysis

SPSS software for Windows version 18.0 (SPSS, Inc, Chicago, Illinois) was used for the statistical analyses. The prevalence of DED was calculated with a 95% confidence interval (CI). The association between DED and potential risk factors was determined with univariate analysis using Pearson’s chi-square test for categorical variables and Student’s t-test for quantitative variables. Multivariate analysis using logistic regression analysis was also performed for the evaluation of the risk factors of DED. *P*-value < 0.05 was considered statistically significant.

### Ethics Statement

This study was approved by the institutional review board (IRB) of the Kangwon National University Hospital and was conformed to the tenets of the Declaration of Helsinki. Informed consent was obtained from all the participants.

## Results

### Prevalence of DED

Of a total of 566 paramedical workers, 232 co-operated with the interview and completed the survey, of whom 35 (15.1%) were male and 197 (84.9%) were female. Prevalence of DED was 42.7% (99/232; 95% CI, 36.6% – 48.7%). Subjects with DED (DED group) showed significantly higher VAS and OSDI score compared to those without DED (non-DED group) (P < 0.001 for both) (Table 3).

**Table 3:** Comparison of the dry eye symptom scores according to the presence and absence of dry eye disease (DED).

Variables	DED group (n = 99)	Non-DED group (n = 133)	*P* value*
Stress visual analog scale (VAS)	16.2 ± 5.8	6.3 ± 5.6	<0.001
Ocular surface disease index (OSDI)	50.7 ± 15.1	25.5 ± 16.4	<0.001

DED group: patients with dry eye disease.

Non-DED group: patients without dry eye disease.

All data are expressed as mean ± SD.

*P value was calculated using Student’s t-test.

### Risk factors for DED

Univariate analysis showed that female sex (P < 0.001), prolonged computer use (*P* = 0.003) and higher stress VAS (*P* < 0.001) and PSS-4 (*P* = 0.009) scores were associated with increased risk of DED. The prevalence of DED was significantly higher in female (47.7%, 94/197) compared to male (14.3%, 5/35) participants (*P* < 0.001). Workers in DED group showed significantly longer duration of computer use(7.9 ± 2.0 hr vs. 7.0 ± 2.8 hr, *P* = 0.003). Both stress VAS (6.7 ± 2.2 vs. 5.6 ± 2.7, *P* < 0.001) and PSS-4 (7.9 ± 2.3 vs. 7.0 ± 2.4, *P* = 0.009) scores were also significantly higher in the DED group compared to non-DED group. However, age, CL wear, history of refractive surgery, hours of paper work, sleep duration, alcohol consumption did not show significant association with (Table 4).

**Table 4:** Univariate analysis of potential risk factors for DED.

Variables	DED group (n = 99)	Non-DED group (n = 133)	*P* value
Age (yr)			
20–29	62 (62.6%)	70 (52.6%)	
30–39	21 (21.2%)	41 (30.8%)	0.227^†^
40 or older	16 (16.2%)	22 (16.5%)	
Sex (M: F)	5.1%: 94.9% (5: 94)	22.6%: 77.4% (30: 103)	<0.001^†^
Contact lens wear	36.4% (36/99)	29.3% (39/133)	0.261^†^
History of refractive surgery	19.2% (19/99)	18.0% (24/133)	0.865^†^
Computer use (hr)	7.9 ± 2.0	7.0 ± 2.8	0.003*
Hours of paper work (hr)	1.2 ± 1.8	0.9 ± 1.4	0.315*
Sleep duration (hr)	6.6 ± 1.1	6.8 ± 1.3	0.193*
Alcohol consumption (g/mo)	257.0 ± 379.5	272.1 ± 393.2	0.769*
Stress score (VAS)	6.7 ± 2.2	5.6 ± 2.7	<0.001*
Stress score (PSS-4)	7.9 ± 2.3	7.0 ± 2.4	0.009

All data are expressed as mean ± SD, or percentage, as appropriate.

*P value was calculated using Student’s t-test.

^†^*P* value was calculated using Pearson’s chi-square test.

Table 5 shows the results of multivariate logistic regression analyses using various models. After adjustment for sex, duration of computer use and stress VAS score, female sex (odds ratio [OR], 4.53; 95% CI, 1.65–12.42; *P* = 0.003) and stress VAS score (OR, 1.17; 95% CI, 1.03–1.32; *P* = 0.013) had significant association with DED (Model 1). After adjustment for sex, duration of computer use and PSS-4 score, female sex (OR, 4.57; 95% CI, 1.67–12.48; *P* = 0.003) and computer use (OR, 1.15; 95% CI, 1.01–1.30; *P* = 0.029) were significant associated to DED (Model 2).

**Table 5:** The results of multivariate logistic regression of potential risk factors for DED.

Variables	Model 1:		Model 2:	
	Adjusted for sex, duration of computer use and stress VAS score		Adjusted for sex, duration of computer use and stress PSS-4 score	
	Odds ratio (95% CI^*^)	*P* value	Odds ratio (95% CI^*^)	*P* value
Sex (female)	4.53 (1.65–12.42)	0.003	4.57 (1.67–12.48)	0.003
Computer use (hr)	1.11 (0.98–1.27)	0.098	1.15 (1.01–1.30)	0.029
Stress score (VAS)	1.17 (1.03–1.32)	0.013		
Stress score (PSS-4)			1.17 (0.99–1.26)	0.127

^*^Confidence interval.

Stress VAS score showed significant correlation with both OSDI (*P* < 0.001, r^2^ = 0.10) and dry eye VAS (*P* < 0.001, r^2^ = 0.16). PSS-4 score also had significant correlation with both OSDI (*P* < 0.001, r^2^ = 0.04) and dry eye VAS (*P* < 0.002, r^2^ = 0.02) score.

## Discussion

In this study, we evaluated the prevalence and risk factors of DED among paramedical workers at a hospital in Korea. The diagnosis of DED was made based on the presence of dry eye symptoms included in the DEQ^2^. Symptom-based definition of DED has been used in the researches of DED worldwide, especially in large population-based studies^1,2,6,23–25^, which might be due to the following reasons: (1) there is no gold standard test of the diagnosis of DED yet^24,25^, (2) Lack of correlation between signs and symptoms of DED has been reported^24–27^, (3) Relief of dry eye symptoms is the main goal of treatment for DED^2,28^, and (4) DEQs were proven to have substantial repeatability^6,24,25,29^. In this study, both OSDI and dry eye VAS were significantly higher in DED group, suggesting the reliability of the DEQ (Table 2).

The present study revealed high prevalence of DED in paramedical workers (42.7%). Prior studies reported the higher prevalence of DED in Asian countries, especially in elderly population^1,2,30^. A study on Japanese elderly population over 60 years reported that the prevalence of DED was 73.5%^30^. Its prevalence in a Korean and Chinese population >65 years was 33.2% and 23.5%, respectively^1,2^. Meanwhile, studies have indicated that DED might be also prevalent in younger age group^6,13,23,31^. Studies on Japanese population reported that the prevalence of DED was over 20% in adolescents and high school students^23,31^. Another Japanese study revealed that the prevalence of DED in young and middle-aged office workers was 27.3% in male and 48.0% in female participants^6^. A study on university students in Ghana also reported a high prevalence of symptom-based DED of 44.3%^32^. These findings suggest that DED may be prevalent in younger population, particularly in office workers and students.

A recent study demonstrated high DED prevalence of 56% in surgical residents with a mean age of 27.8 years old, and suggested that working inside the operating room, in which the ventilation environment is closed and precise procedures with great concentration are performed, might increase the risk of DED^13^. These findings, along with our results, suggest that hospital worker might have increased risk of DED^13^. The environmental characteristics of hospital, such as, low indoor humidity, reduced indoor air flow and exposure to volatile organics might make individuals more prone to develop DED^33,34^. Makateb *et al*.^35^ revealed that night-time working were associated with decreased tear film stability and worsening of dry eye symptoms. Thus, it can be postulated that frequent night-shift in hospital workers may increase the risk of DED. There findings suggest that improvement of hospital environment and reduction in night shift work could lower the risk of DED in paramedical workers. Further studies are needed to evaluate the nature and risk factors of DED in paramedical workers.

The results showed that stress VAS was associated with an increased risk of DED. PSS-4 score also had significant association with DED in univariate analysis, although no significant association was found in multivariate analysis. These findings suggest a close relationship between psychologic stress and DED. Although the association between DED and psychiatric conditions including depression and post-traumatic stress disorder have been reported by several studies^10,36,37^, there has been only few studies regarding the association between psychological stress and DED, probably due to the difficulty in the measurement of the psychological stress. We measured the psychologic stress among the paramedical workers using the stress VAS because it was proven to be a simple, suitable and efficient tool for the assessment of psycholgical stress by occupational physicians^20,21^. We also used PSS-4 because it was also shown to be a reliable tool in quantification of perceived psychological stress^22^. Na *et al*.^11^ also demonstrated that DED was associated with an increased risk of severe psychological stress, depression and anxiety in Korean women^11^. These findings, along with our results. suggest that psychological stress may be associated with an increased risk of DED. Therefore, it can be postulated that management of psychological stress can reduce the risk of DED in hospital workers. Although the pathophysiology underlying the association between psychological stress and DED is still ulcer, several mechanisms can be postulated, as follows: (1) Psychological stress may affect pain perception, and make individuals with increased stress more prone to feel dry eye symptoms. Geva *et al*.^38^ revealed that acute stress can reduce the ability to modulate pain. Cohen *et al*.^39^ also suggested that psychological stress can increase pain by inducing elevation of cortisol level. (2) Psychological stress can increase systemic inflammatory activity by promoting production of inflammatory cytokines^40^, which lead to increased risk of developing various diseases, including DED^41,42^. (3) Perceived stress was shown to be associated with of somatization^43^. Thus, somatization induced by psychological stress can aggravate dry eye symptoms. (4) Psychological stress can result in depression^40^, which is a well-known risk factor for DED^10,36,37^. (5) There is a possibility that the effect of Korean culture, such as, pressure for high achievement, vigorous competition for jobs and promotion and emotional stress from co-workers and customers might also play a role. We believe further studies including larger population are necessary to evauate the pathophysiology underlying the association between the two conditions.

This study showed that female sex was associated with DED, which is in agreement with the results of the previous studies^1,2,6,12^. Vehof *et al*.^44^ revealed that women showed higher dry eye symptom scores than men and increased discrepancy between dry eye signs and symptoms. Schaumberg *et al*.^45^ demonstrated that women tended to experience more severe and frequent DED symptoms than men, and also complained a greater influence of DED on daily activities. A recent study by Na *et al*.^46^ also reported that menstrual irregularity had an association with DED. These findings suggest that sex hormones might affect ocular surface environment through their effects on corneal sensitivity, conjunctival goblet cells, lacrimal glands and Meibomian glands^1,2^. Japanese studies revealed increased prevalence of DED in female sex in Japanese high school students and young office workers^6,23^. Our results also showed high prevalence of DED in young female paramedical workers, indicating that sex hormones might play a role in DED in young women as well as older ones. However, the reason for increased risk of DED in the female population is still unclear, and therefore further studies are warranted.

The results also suggest that long duration of computer use was possibly associated with an increased risk of DED, which is in consistent with the results of the prior studies that prolonged use of video display terminal (VDT) was associated with DED^6,12^. Kawashima *et al*.^47^ revealed that 60% of the workers using VDT had DED, which might cause a significant impairment of the productivity of the workers^48^. Prolonged VDT use may be associated with decreased blinking rates and increased tear evaporation, which can lead to tear film instability and hyperosmolarity, and eventually short break-up time type DED^6,48^. Moreover, blue light emission from VDTs can suppress the synthesis of melatonin, particularly in young population^31^. Decrease in melatonin level might lead to disruption of sleep cycle, which can aggravate dry eye symptoms^49^.

The present study has limitations as follows: (1) Data of only 232 participants from only one hospital were included in this study, which is substantially small number to represent the population of paramedical workers in Korea. However, we still believe this study can provide significant information regarding the characteristics of DED in paramedical workers, particularly because it revealed an association between DED and psychological stress. We also believe further studies including larger population group in multiple hospitals are needed to evaluate the characteristics of DED in paramedical workers. (2) Pathophysiology underlying the association between stress and DED is still unclear, and the correlation analysis could not show causative relationship, which necessitate further studies. (3) Diagnosis of DED was made only based on the presence of dry eye symptoms, and examination for dry eye signs was never performed. However, symptom-based approach using DEQs was proven to be highly reliable for identification of DED^29^. A number of previous studies were successfully conducted using the symptom-based definition of DED using DEQs^1,2,6,23–25,28^. Nevertheless, we still believe that ophthalmologic examination should be incorporated into study protocols for elucidation of the nature of DED in further studies with larger population.

In conclusion, this study showed that DED may be prevalent in paramedical workers in Korea. Psychological stress measured using VAS had significant association with a risk of DED. Female sex was also associated with an increased risk of DED, and prolonged computer use was also possibly associated with DED.

## Data Availability

All the data supporting the conclusions of this article is included in the present article.
